# Predictive Value of Brain Volumetry in the Response to Subthalamotomy Using High-Intensity Focused Ultrasound Treatment in Patients With Parkinson's Disease

**DOI:** 10.1155/padi/8780938

**Published:** 2025-06-12

**Authors:** Alfaro-Nasta E., Gonzalez-Mendez P., Lorenzoni J., Juri C., Andia M.E.

**Affiliations:** ^1^Morphology Department, School of Medicine, Universidad del Desarrollo, Santiago, Chile; ^2^Millennium Institute for Intelligent Healthcare Engineering–iHEALTH, Santiago, Chile; ^3^Doctoral Program in Medical Science, School of Medicine, Pontificia Universidad Católica de Chile, Santiago, Chile; ^4^Neurosurgery Department, School of Medicine, Pontificia Universidad Católica de Chile, Santiago, Chile; ^5^Neurology Department, School of Medicine, Pontificia Universidad Católica de Chile, Santiago, Chile; ^6^Radiology Department and Biomedical Imaging Centre, School of Medicine, Pontificia Universidad Católica de Chile, Santiago, Chile

**Keywords:** brain volumetry, FreeSurfer, high-intensity focused ultrasound, MRg-FUS, Parkinson's disease, subthalamotomy

## Abstract

**Objective:** Subthalamotomy using magnetic resonance-guided high-intensity focused ultrasound (MRg-FUS) is a noninvasive therapy that improves the cardinal symptoms of Parkinson's disease. However, clinical outcomes show high variability even when all inclusion criteria for this treatment are met. In this work, we aim to study the relationship between brain volumetry and clinical response in patients undergoing this treatment.

**Methods:** Twenty patients who underwent MRg-FUS subthalamotomy were included and evaluated at baseline and 3 months post-treatment. Brain volumes were obtained from pretreatment MRI scans processed with the open-source package FreeSurfer. The treatment response was assessed using the movement disorder society unified Parkinson's disease rating scale (MDS-UPDRS). Principal component analysis (PCA) and clustering methods were used to identify groups of patients with similar clinical outcome.

**Results:** PCA identified 2 clusters of patients, and a sensitivity and a specificity of 80% classified patients who will have a response to treatment with an improvement greater than 40% of the pretreatment UPDRS III scale. A positive association was found with the response to treatment with the variables: brain volume, cortical thickness and volume of total gray matter, and subcortical gray matter and white matter. On the other hand, a negative association was found with the response to treatment with the variables: ventricular volume.

**Conclusion:** Our findings suggest that brain atrophy, reduced global cortical thickness, and increased ventricular volume are significantly associated with the predicting treatment response in Parkinson's disease patients undergoing MRg-FUS subthalamotomy.

## 1. Introduction

Parkinson's disease (PD) is a progressive neurodegenerative disorder affecting approximately 6 million people worldwide, and this number is expected to double in the next 20–30 years [1, 2]. Some of the described pathogenic mechanisms include neuroinflammation, alterations in α-synuclein metabolism, calcium metabolism, oxidative stress, and mitochondrial dysfunction [3]. The main pathological features of PD include the accumulation of Lewy bodies and the loss of dopaminergic neurons in the substantia nigra pars compacta. The reduction in dopamine disrupts the functioning of the cortico-striato-thalamo-cortical circuit, leading to the cardinal motor symptoms of the disease, such as bradykinesia, freezing, rigidity, and resting tremor. In addition to motor symptoms, nonmotor manifestations such as depression, anxiety, anosmia, dysautonomia, and sleep disorders may also occur [3, 4]. Currently, there is no cure for PD, and clinical treatment is symptomatic, aimed at improving the quality of life of patients.

Currently available symptomatic treatments can be divided into pharmacological and nonpharmacological approaches. The pharmacological approach aims to mainly increase dopamine activity through precursors, agonists, or other agents. However, as the disease progresses, medications gradually lose their effectiveness. It is estimated that within 3–5 years, patients develop treatment-related complications, although the timeframe can vary [5]. The second group of therapies involves a surgical approach, including techniques such as deep brain stimulation (DBS) [6] and magnetic resonance-guided high-intensity focused ultrasound (MRg-FUS) [7].

Neuroimaging has gained a prominent role in recent years in the diagnosis, prognosis, and treatment of various pathologies of the nervous system. Magnetic resonance imaging (MRI) is a technique of choice for this purpose due to its sensitivity and safety. MRI images allow for the assessment of morphological and functional changes in brain structures. Several software packages have been developed to facilitate pre- and postprocessing and quantitative morphological analysis of these images, including FreeSurfer and FSL. FreeSurfer provides a range of algorithms to quantify the brain's functional, connectivity, and structural properties, while FSL is a comprehensive library of analysis tools for functional and diffusion MRI brain imaging data [8–10].

The therapeutic use of brain lesions for movement disorders has re-emerged in recent years by the appearance of the MRg-FUS technique as a less invasive and more precise treatment alternative than DBS for patients who prefer not to undergo invasive surgery [11]. The procedure is guided by MRI and produces a therapeutic lesion through thermo-ablation using ultrasound. The neurosurgical targets are similar to those of DBS in PD, such as the ventral intermediate nucleus of the thalamus, subthalamic nucleus, and internal globus pallidus [12]. MRg-FUS thalamotomy is accepted for the treatment of patients with refractory tremor in PD, while subthalamotomy and pallidotomy have been primarily used in patients with asymmetric PD to treat rigidity, bradykinesia, and tremor and/or patients with motor complications, such as L-dopa-induced dyskinesias [12].

MRg-FUS technology has proven effective in clinical trials; however, there is variability in clinical outcomes [13]. Efforts have been made to identify neuroimaging markers that can predict clinical outcomes and treatment effects. Routine MRI images used in clinical practice (T1, T2, and diffusion) can be valuable for assessing characteristics that may explain variability in the MRg-FUS treatment response.

Volumetric markers measured by MRI have been studied in various neurological pathologies. Schirmer et al. [14] demonstrated the predictive value of brain volume in poststroke recovery. Lomer et al. [15] showed predictive value regarding progression and cognitive decline in multiple sclerosis, and Anderl-Straub et al. [16] demonstrated this in frontotemporal dementia and Koscik in spinocerebellar ataxia type 1 [17].

Based on the above and considering that MRg-FUS subthalamotomy is an irreversible treatment and there is still variability in the clinical response, it is necessary to advance in improving the selection of patients who have better chances of a successful outcome. In the present work, we aim to study the capacity of brain volumetry measured in pretreatment brain MRI to predict the clinical response to MRg-FUS subthalamotomy in PD patients.

## 2. Patients and Methods

### 2.1. Study Population and Neurological Evaluation

Patients were selected from the database of those who underwent subthalamotomy with MRg-FUS between 2022 and 2023 at the Clinica San Carlos in Red Salud UC-Christus and the neurology department of the Pontificia Universidad Católica de Chile. The inclusion criteria used were as follows: availability of precontrast whole brain volumetric T1w MRI images and availability of clinical assessments of part III of the movement disorder society unified Parkinson's disease rating scale (MDS-UPDRS) pre- and 3 months post-treatment.

The MDS-UPDRS scale includes four parts: Part I assesses nonmotor characteristics of daily life; Part II involves motor characteristics of daily life; Part III corresponds to motor examination; and Part IV records motor complications. The Part III of the scale was used as clinical outcome since MRg-FUS subthalamotomy seeks to improve the cardinal motor alterations of PD. The score associated with this part of the evaluation ranges from 0 to 108. All patients were evaluated OFF medication by a movement disorder specialist.

Clinical data were collected, including sex, age, age at diagnosis, disease duration, hemisphere of intervention, and baseline and 3-month MDS-UPDRS part III scores. All patients met the inclusion criteria defined by the movement disorders unit of the Pontificia Universidad Católica de Chile UC-Christus Health Network to undergo MRg-FUS therapy.

This study was approved by the Scientific Ethics Committee of the Pontificia Universidad Católica de Chile, ID 230503020, and all participants provided written informed consent before their inclusion in the study.

### 2.2. MRI Acquisition Protocol

All magnetic resonance images were obtained using a GE Healthcare SIGNA Artist 1.5T scanner with a 24-channel head coil. The clinical protocol included a T1-weighted volumetric sequence using 3D gradient echo, TR/TE: 8.4/3.1 ms, matrix size: 240 × 240, flip angle: 8°, number of slices: 248, and isotropic voxel: 1 × 1 × 1 mm^3^.

### 2.3. Subthalamotomy MRg-FUS

The subjects included had their heads shaved and were placed under conscious sedation. A stereotactic frame was attached to the head to allow precise targeting of the treatment site. The patient's head was fixed to the MRI table and covered with a membrane through which refrigerated water circulated constantly. An initial brain MRI was obtained, and the subthalamic nucleus was located using pre-established stereotactic coordinates. Sonication was performed to calibrate and verify clinical effects at temperatures of approximately 46°C and 50°C, respectively. Once beneficial effects were confirmed and adverse effects ruled out, lesions were created at approximately 58°C at the three pre-established targets in the subthalamic nucleus and pallidothalamic tract [18].

### 2.4. Evaluation of Brain Volumes and Cortical Thickness

For the purposes of this study, cerebral gray matter includes the volume of the cerebral cortex (and average cortical thickness), subcortical gray matter volume, and total gray matter volume. Segmentation of cerebral gray and white matter volumes, as well as subcortical structures, was performed using the open-source software package FreeSurfer (version 7.4.1, https://surfer.nmr.mgh.harvard.edu/). The segmentation process was conducted on the T1w volumetric MRI image [19, 20].

The data obtained from FreeSurfer that was used for statistical analysis include the following: (1) average cortical thickness (mm), (2) brain volume (mm^3^), (3) ventricular volume (mm^3^), (4) white matter volume (mm^3^), (5) subcortical gray matter volume (mm^3^), (6) total gray matter volume (mm^3^), (7) choroid plexus (mm^3^), and (8) estimated total intracranial volume (eTIV, mm^3^). The volumes obtained were adjusted for the eTIV, providing adjusted volumes for each of the studied brain areas. Cortical thickness was not adjusted, as justified by previous studies [21, 22]. The adjusted volumes used in this work were selected based on previous studies that related them to the diagnosis, progression, response to treatment, and search for predictive markers of various degenerative brain pathologies [23–25].

### 2.5. Statistical Analysis

Data were expressed as mean ± standard deviation or as median (Q1, Q3). The Shapiro–Wilk test was applied to assess data normality, and nonparametric tests were used for data that did not follow a normal distribution. Principal component analysis (PCA) and the KNN algorithm were performed to reduce data dimensionality and identify clusters of similar populations. Comparisons of the clusters identified in the PCA were conducted using the Mann–Whitney *U* test, with statistical significance set at *p* ≤ 0.05. To evaluate the predictive capacity of the clusters identified by PCA to predict the response to HIFU treatment, we performed a multivariate logistic regression analysis with backward elimination. All analyses were performed using the statistical software IBM SPSS Statistics v. 29.0.2.0 and the R statistical package Version 4.0.2.

## 3. Results

A total of 50 PD patients who received the MRg-FUS subthalamotomy treatment were identified. 28 of whom had T1w volumetric MRI studies in their preoperative evaluation, and from them, 20 have clinical assessments of part III of the MDS-UPDRS pre- and 3 months post-treatment.

The demographic data of the 20 patients included in the study are shown in Table 1.

**Figure 1:**
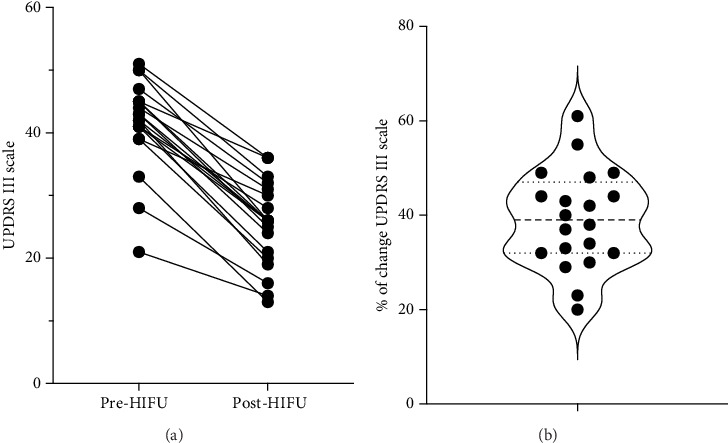
(a) UPDRS III scale assessment of patients pre- and post-treatment with MRg-FUS. (b) Distribution of the percentage improvement in the UPDRS III scale pre- and post-treatment with MRg-FUS.

All patients showed improvement after treatment as assessed by the UPDRS III scale post-treatment (Figure 1(a)).  Figure 1(b) illustrates the distribution of percentage change on the UPDRS III scale pre- and post-treatment, with a global median change of 40%. For the purpose of this work, successful treatment was defined as an improvement equal or larger than 40% of change in the Part III of the MDS-UPDRS scale pre and post treatment.

Table 2 shows the quantification of adjusted brain volumetry, including brain volume, brain volume excluding ventricles, ventricular volume, white matter brain volume, subcortical gray matter volume, and total gray matter volume for the 20 patients.

Table 3 list the brain volumetry variables used in the dimensionality reduction analysis with PCA and the following cluster analysis with KNN.

**Figure 2:**
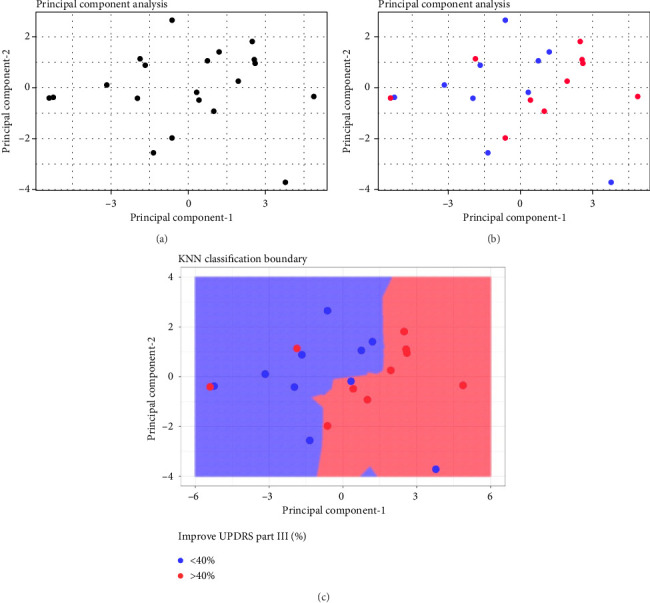
(a) Result of principal component analysis (PCA) using Components 1 and 2, (b) patient labeling based on treatment effectiveness greater than or less than 40%, and (c) result of KNN classification and identification of two clusters.

Figure 2(a) shows the principal components 1 and 2 for the 20 patients; Figure 2(b) labels each patient based of the clinical outcome (successful: improvement equal or larger than 40% of change in the Part III of the MDS-UPDRS scale pre- and post-treatment).  Figure 2(c) shows the two independent clusters identified by the KNN algorithm. Cluster 1 mainly groups patients with a lower response to treatment and contains 8 true negative patients and 2 false negative patients. Cluster 2 mainly groups patients with a better response to treatment and contains 8 true positive patients and 2 false positive patients. In global, the cluster analysis showed a sensitivity of 80% and a specificity of 80% for predicting a treatment response with improvement greater than 40% on the pretreatment UPDRS III scale.

To better understand the characteristics of the patients in each group, Table 4 compares the main demographic and brain volume values of both clusters.

**Figure 3:**
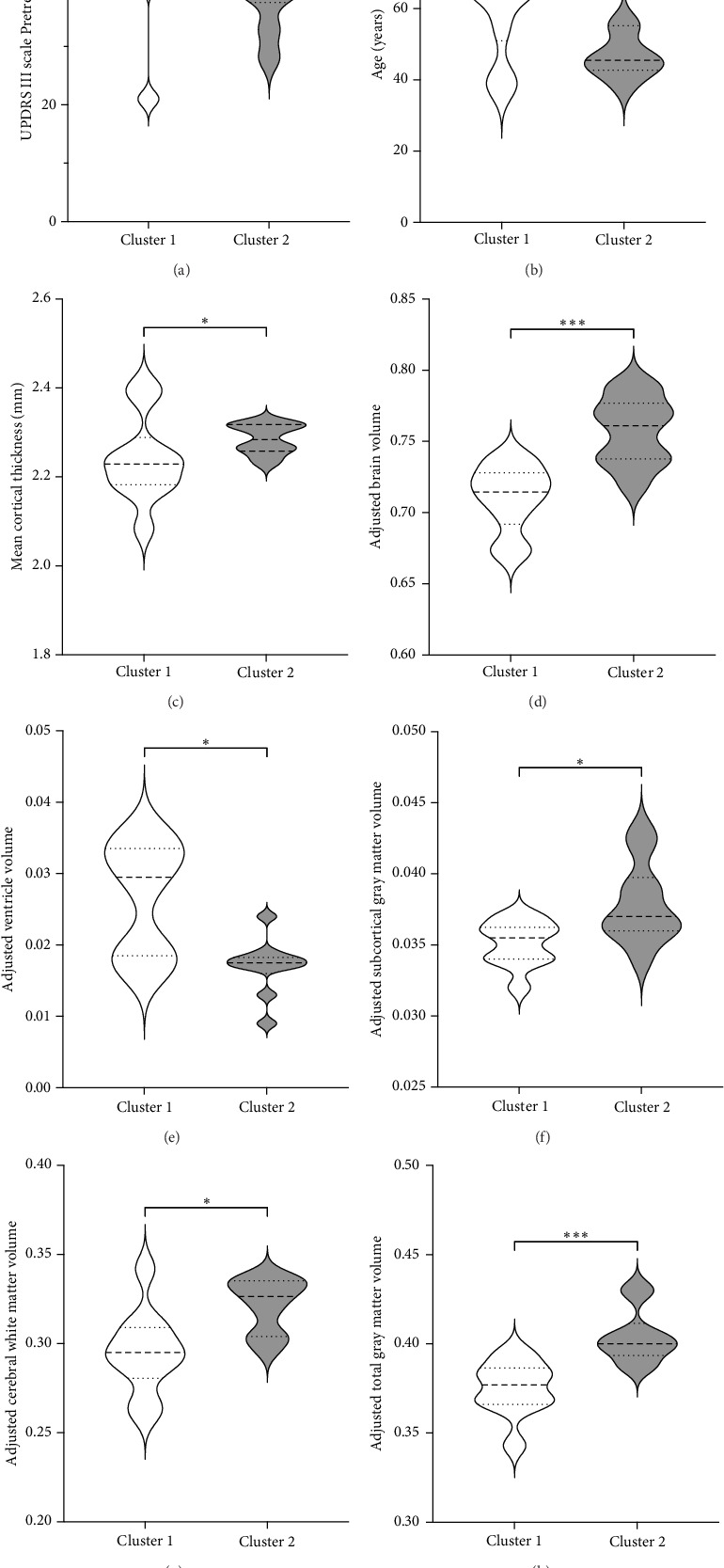
Comparison between Cluster 1 and Cluster 2 with respect to statistically significant variables.

Figure 3 shows the comparison between main characteristics of both clusters. Cluster 1, which had a lower response to treatment, had a mean age of 61.5 years and a mean reduction in the UPDRS scale of 14.4 points. In contrast, cluster 2, which responded significantly better to treatment, had a mean age of 48.8 years with a reduction of 17.8 points in the UPDRS III scale; the age difference between both clusters did not reach statistical significance. Patients from both clusters did not present significant differences in the pretreatment score of Part III of the MDS-UPDRS. Interestingly, parameters associated with cerebral atrophy showed significant differences between clusters. Total brain volume was significantly larger in patients from Cluster 2, who had a better treatment response. Similarly, Cluster 2 had significantly lower ventricular volume, greater total white matter volume, greater subcortical gray matter volume, greater total gray matter volume, and greater average cortical thickness compared to Cluster 1.

No significant differences were found regarding disease duration, hemisphere of intervention, choroid plexus volume, eTIV, and the ratio of the volume of the intervened hemisphere to the nonintervened hemisphere.

**Figure 4:**
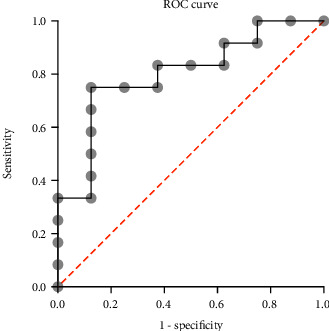
ROC curve of the predictive analysis of the treatment response using the cluster identified by PCA.

For the multivariate logistic regression analysis, we used the following predictor variables: Cluster identified by PCA, Part III of the MDS-UPDRS pretreatment, age, and time since diagnosis. Using the backward elimination method, the only statistically significant predictor variable identified was the cluster identified by PCA, with an OR: 16.3, CI: 95% (1.4, 801.6), an AUC of 0.8021, CI: 95% (0.6, 1.0), *p* = 0.025 (Figure 4).

## 4. Discussion

This study was exploratory and retrospective. Although the sample size is relatively small, it represents a Latin American patient population not previously represented in studies in this area, making this study the first of its kind.

By characterizing anatomical and volumetric features of the brain using automated methods, we sought to find associations between morphological characteristics and clinical response to subthalamotomy by MRg-FUS. We demonstrated that there were volumetric characteristics of the patients' gray and white matter that PCA analysis was able to cluster into two groups; and more importantly, these two clusters were an excellent predictor of the treatment response, with an OR of 16.3.

Previous studies have linked brain structure volumes to the clinical response in various neurodegenerative diseases. For instance, Saha et al. [26] evaluated the correlation between the transcranial stimulation response in Alzheimer's disease and the volumetry of gray and white matter in the right and left dorsolateral prefrontal cortex. Müller et al. [27] related basal forebrain volume to the cognitive response to galantamine in elderly healthy patients.

The distribution by sex and age of disease onset aligns with a higher frequency in men than women [28]. Regarding age, although it did not reach statistical significance, there was a tendency that younger patients has a better response to treatment, this could be explained by the lower accumulated brain atrophy associated with age and that in the first years of the disease there is accelerated atrophy which would affect brain volumes and plasticity, which deteriorates the ability to respond to pharmacological and surgical treatment. Aygun et al. [29], studying the levodopa response as a predictor of DBS effectiveness, found that older patients had a poorer response to levodopa, making them less suitable candidates for DBS. Similar findings were reported by Du et al. [30] regarding levodopa, with poorer responses related to bradykinesia and general PD signs in older subjects.

Changes in brain morphology and the brainstem have been associated with the pathophysiology of PD, motor/nonmotor symptoms, and disease progression [31]. In our study, we found a significant association of total gray and white matter volume, positively correlated, and ventricular volume, negatively correlated, with a better treatment response [32].

Gray matter atrophy has been used as a predictor of progression and severity of motor symptoms [33]. The difference in cortical thickness between clusters may be explained by the accumulated atrophy in Cluster 1, which, although not showing significant differences in years with the disease. The volume of subcortical gray matter presents variable evidence, supporting the idea that a global measurement of subcortical gray matter may be more representative of the degree of atrophy. This aligns with findings by Lewis et al. [34], who found a general decrease in subcortical nuclei in patients with more motor symptoms and discrepancies in atrophy degrees of the putamen, caudate, and globus pallidus compared to healthy controls. Despite the variability in isolated measurements of basal nuclei and thalamus, we demonstrated that in patients with a better treatment response, a smaller volume of subcortical gray matter is associated with a lower likelihood of treatment success. Our findings suggest that total gray matter volume may be considered a marker of disease progression and treatment response.

Regarding ventricular volume, our findings are consistent with those reported by Younce et al. [35], who, studying markers of the response to STN DBS, found that larger ventricular volume was associated with poorer improvement in motor symptoms. He also demonstrated an association with thalamic volume, where better-preserved thalamic volume was associated with a better treatment response. A decrease in thalamic volume is a sign of atrophy, which can lead to increased ventricular volume. Younce did not find significance in cortical thickness related to the treatment response, which contrasts with our study's findings. This discrepancy may be explained by the narrower age range and disease duration in Younce's study, resulting in similar degrees of atrophy in that group.

Measurement of white matter volume and hyperintensities has gained importance as markers of damage, disease progression, and treatment response [36, 37]. Studies such as Sara Pietracupa's [38], which aimed to identify abnormalities in gray and white matter in early PD patients and relate them to motor and nonmotor signs, found lower fractional anisotropy and higher mean, axial, and radial diffusivity in most white matter tracts (corticospinal tracts, internal and external capsules, thalamic radiations, knee and body of the corpus callosum, cerebellar peduncles, and superior and inferior longitudinal and fronto-occipital fascicles). It also demonstrated that white matter deterioration preceded gray matter deterioration. Agosta et al. [39], studying brain topography in patients with different stages of PD, found that white matter degeneration continued and became more pronounced as the disease progressed, while cortical atrophy accelerated from mild to moderate stages and then stabilized, continuing at a slower rate. Rektor et al. [40], studying early PD patients, reported similar results regarding white matter degeneration preceding gray matter damage in patients with motor signs and normal cognition. These findings align with our study regarding white matter atrophy and cortical thickness. Considering this, we can infer that better-preserved white matter volume could serve as a predictor of treatment success. This is consistent with Andrews et al. [41], who found better treatment responses in DBS patients with larger white matter volumes and smaller ventricular volumes.

## 5. Conclusion

Our findings suggest that the brain atrophy markers evaluated in this study are statistically significant in predicting a better response to treatment in patients undergoing subthalamotomy by MRg-FUS. The classification method derived from PCA analysis identified two patient clusters, and logistic regression analysis demonstrated that these clusters allow estimating the response to treatment, which could become a useful clinical tool for patient selection. The brain volume measurements used in our predictive model can be obtained from routine imaging exams and processed with automated quantification methods, indicating a strong potential for clinical translation. However, our results should be interpreted with caution due to the small sample size and the fact that the study was conducted at a single healthcare institution and population group. Larger prospective studies are needed to confirm these results.

## Figures and Tables

**Table 1:** PD patients' demographic data and PD evaluation.

	*N* total	Mean	SD	Range
Sex (M/F)	16/4			
Age (years)	20	55.20	12.80	(38–76)
Time from diagnosis (years)	20	7.35	2.92	(2–13)
UPDRS III before treatment	20	41.50	7.26	(21–51)

**Table 2:** Brain volumetry quantification adjusted by estimated total intracranial volume (e-TIV).

ID	Mean cortical thickness (mm)	Adjusted brain volume	Adjusted brain no ventricle volume	Adjusted ventricle volume	Adjusted cerebral white matter volume	Adjusted subcortical gray matter volume	Adjusted total gray matter volume	Choroid plexus (mm^3^)	Estimated total intracranial volume
P_1	2.405	0.737	0.717	0.019	0.312	0.036	0.391	1269	1,789,140
P_2	2.314	0.789	0.780	0.009	0.335	0.043	0.428	1326	1,495,490
P_3	2.317	0.767	0.748	0.018	0.336	0.039	0.395	1345	1,707,820
P_4	2.319	0.740	0.724	0.017	0.304	0.039	0.405	1295	1,532,161
P_5	2.230	0.770	0.753	0.018	0.332	0.036	0.404	1507	1,716,108
P_6	2.268	0.739	0.720	0.019	0.318	0.037	0.385	1583	1,417,354
P_7	2.186	0.710	0.693	0.016	0.308	0.034	0.368	1515	1,614,237
P_8	2.171	0.697	0.666	0.031	0.267	0.037	0.385	1486	1,526,716
P_9	2.322	0.773	0.749	0.024	0.337	0.034	0.399	1682	1,765,650
P_10	2.228	0.724	0.692	0.032	0.299	0.034	0.380	1230	1,481,531
P_11	2.257	0.719	0.699	0.02	0.289	0.036	0.395	1275	1,255,672
P_12	2.246	0.671	0.638	0.033	0.260	0.032	0.365	1318	1,313,948
P_13	2.084	0.676	0.640	0.036	0.285	0.034	0.343	790	1,499,351
P_14	2.383	0.725	0.690	0.035	0.293	0.037	0.382	915	1,824,604
P_15	2.262	0.788	0.771	0.017	0.325	0.042	0.431	1010	1,223,519
P_16	2.298	0.755	0.741	0.013	0.328	0.036	0.400	1186	1,657,342
P_17	2.230	0.710	0.682	0.028	0.297	0.035	0.372	1588	1,680,276
P_18	2.270	0.734	0.717	0.017	0.304	0.036	0.398	1235	1,557,844
P_19	2.246	0.719	0.700	0.018	0.296	0.037	0.389	1118	1,404,307
P_20	2.188	0.738	0.720	0.017	0.342	0.036	0.365	1691	1,618,763

**Table 3:** Clinical and brain volumetry variables used in the dimensionality reduction analysis.

• Sex and age	• Total brain volume except ventricular volume	• Subcortical gray matter volume
• Time since diagnosis	• Total brain gray matter volume	• Estimated total intra cranial volume
• Part III of the MDS-UPDRS pretreatment	• Plexus choroid volume	• Right hemisphere volume
• Total brain volume	• Cerebral white matter volume	• Left hemisphere volume
• Ventricular volume	• Mean cortical thickness	• Rate of the intervened versus nonintervened hemisphere volume

**Table 4:** Comparison of demographic and volumetric characteristics between Cluster 1 (UPDRS III improvement < 40%) and Cluster 2 (UPDRS III improvement > 40%).

	Cluster 1	Cluster 2
Sex (M/F)	9/1	7/3
Age (years)	61.5 (13.12)	48.8 (9.27)
PD time from diagnosis (years)	7.20 (2.53)	7.50 (3.41)
Intervened hemisphere (right/left)	5/5	4/6
Mean cortical thickness (mm)	2.238 (0.10)^∗^	2.285 (0.03)
Adjusted brain segmentation without ventricle volume	0.684 (0.03)^∗^	0.740 (0.03)
Adjusted ventricle volume	0.027 (0.01)^∗^	0.017 (0.004)
Adjusted cerebral white matter volume	0.295 (0.02)^∗^	0.322 (0.02)
Adjusted subcortical gray matter volume	0.035 (0.002)^∗^	0.038 (0.003)
Adjusted total gray matter volume	0.375 (0.02)^∗^	0.404 (0.02)
Adjusted choroid plexus (mm^3^)	1308 (285)	1329 (210)
Estimated total intracranial volume (mm^3^)	1,560,424 (184,836)	1,547,760 (169,718)
Right hemisphere volume	0.295 (0.02)^∗^	0.320 (0.01)
Left hemisphere volume	0.296 (0.01)^∗^	0.323 (0.01)
Rate hemisphere intervened versus not intervened	0.997 (0.02)	1.002 (0.01)

*Note:* Data are presented as mean and standard deviation.

^∗^Significant difference *p* ≤ 0.05.

## Data Availability

The data that support the findings of this study are available on request from the corresponding author. The data are not publicly available due to privacy or ethical restrictions.
